# One‐locus‐several‐primers: A strategy to improve the taxonomic and haplotypic coverage in diet metabarcoding studies

**DOI:** 10.1002/ece3.5063

**Published:** 2019-03-18

**Authors:** Emmanuel Corse, Christelle Tougard, Gaït Archambaud‐Suard, Jean‐François Agnèse, Françoise D. Messu Mandeng, Charles F. Bilong Bilong, David Duneau, Lucie Zinger, Rémi Chappaz, Charles C.Y. Xu, Emese Meglécz, Vincent Dubut

**Affiliations:** ^1^ Aix Marseille Univ, Avignon Univ CNRS, IRD, IMBE Marseille France; ^2^ Agence de Recherche pour la Biodiversité à la Réunion (ARBRE) Saint‐Leu, La Réunion France; ^3^ ISEM, CNRS, IRD, EPHE Université de Montpellier Montpellier France; ^4^ Irstea, Aix Marseille Univ, RECOVER Aix‐en‐Provence France; ^5^ Laboratory of Parasitology and Ecology, Departement of Animal Biology and Physiology University of Yaoundé I Yaoundé Cameroon; ^6^ Université Toulouse 3 Paul Sabatier CNRS, ENSFEA, EDB (Laboratoire Évolution & Diversité Biologique) Toulouse France; ^7^ Institut de Biologie de l'Ecole Normale Supérieure (IBENS), Ecole Normale Supérieure, CNRS, INSERM PSL Research University Paris France; ^8^ Redpath Museum and Department of Biology McGill University Montréal Quebec Canada

**Keywords:** cytochrome *c* oxidase subunit I gene, diet analysis, eDNA, false negatives, metabarcoding, PCR primers

## Abstract

In diet metabarcoding analyses, insufficient taxonomic coverage of PCR primer sets generates false negatives that may dramatically distort biodiversity estimates. In this paper, we investigated the taxonomic coverage and complementarity of three cytochrome *c* oxidase subunit I gene (COI) primer sets based on in silico analyses and we conducted an in vivo evaluation using fecal and spider web samples from different invertivores, environments, and geographic locations. Our results underline the lack of predictability of both the coverage and complementarity of individual primer sets: (a) sharp discrepancies exist observed between in silico and in vivo analyses (to the detriment of in silico analyses); (b) both coverage and complementarity depend greatly on the predator and on the taxonomic level at which preys are considered; (c) primer sets’ complementarity is the greatest at fine taxonomic levels (molecular operational taxonomic units [MOTUs] and variants). We then formalized the “one‐locus‐several‐primer‐sets” (OLSP) strategy, that is, the use of several primer sets that target the same locus (here the first part of the COI gene) and the same group of taxa (here invertebrates). The proximal aim of the OLSP strategy is to minimize false negatives by increasing total coverage through multiple primer sets. We illustrate that the OLSP strategy is especially relevant from this perspective since distinct variants within the same MOTUs were not equally detected across all primer sets. Furthermore, the OLSP strategy produces largely overlapping and comparable sequences, which cannot be achieved when targeting different loci. This facilitates the use of haplotypic diversity information contained within metabarcoding datasets, for example, for phylogeography and finer analyses of prey–predator interactions.

## 1 INTRODUCTION

Diet studies are critical to the understanding of species interactions, trophic structures, and trophic dynamics (Nielsen, Clare, Hayden, Brett, & Kratina, 2018). They have been applied to a vast set of issues in ecology, evolution, and conservation, such as predator/prey interactions and habitat use (Corse et al., 2010; Sánchez‐Hernández, 2014), trophic niche partitioning (Kartzinel et al., 2015; Trevelline et al., 2018), and the delineation of habitats for guiding species conservation (Quéméré et al., 2013), management (Chivers et al., 2013), and habitat restoration (Motte & Libois, 2002). Diet studies have also proved critical in interfacing agriculture and ecology by assessing the effects of agricultural practices or policies on the trophic behaviors of species (Llaneza & López‐Bao, 2015; Mollot et al., 2014) and by evaluating the ecosystem services of wild species, such as in the control of crop pests (Aizpurua et al., 2018; McCracken et al., 2012).

Over the last decade, considerable efforts have been made toward improving diet assessment methods, in particularly those based on high‐throughput sequencing (HTS) and DNA metabarcoding on environmental samples (Taberlet, Bonin, Zinger, & Coissac, 2018; Taberlet, Coissac, Pompanon, Brochmann, & Willerslev, 2012). DNA metabarcoding has been demonstrated as an efficient alternative to traditional methods of diet analysis (e.g., morphological gut content or stable isotope analyses) by improving both the accuracy and taxonomic resolution of prey identifications, as well as the detection of soft‐bodied, small, or rare prey (Clare, 2014; McInnes et al., 2017; Nielsen et al., 2018; Pompanon et al., 2012). While these advances have considerably enlarged our ability to study large‐scale and highly‐resolved trophic networks (Clare, 2014; Evans, Kitson, Lunt, Straw, & Pocock, 2016; Roslin & Majaneva, 2016), they still suffer from a number of methodological issues (reviewed in Alberdi et al., 2019). In particular, false positives and negatives are common in metabarcoding datasets (Alberdi, Aizpurua, Gilbert, & Bohmann, 2018; Corse et al., 2017; Piñol, Mir, Gomez‐Polo, & Agustí, 2014; Taberlet et al., 2018). False positives correspond to experimental/molecular artifacts (e.g., PCR errors, tag switching, or cross‐sample contaminations) leading to the detection of taxa that were not initially present in the sample. Several experimental and bioinformatic procedures based on negative and positive controls and on technical replicates were developed to filter out such artifacts (Alberdi et al., 2018; Corse et al., 2017; Galan et al., 2018). False negatives correspond to taxa that are not detected while being present in the sample. Although false negatives often occur for rare taxa (e.g., Ficetola et al., 2015), they are also produced when the affinity between primer and primer‐binding sites during polymerase chain reaction (PCR) is low (e.g., Elbrecht & Leese, 2017a; Vamos, Elbrecht, & Leese, 2017), which in turn will determine amplification success and hence the taxonomic coverage of a given primer set. Although there is growing use of control samples to assess levels of false positives (De Barba et al., 2014; Beng et al., 2016; Corse et al., 2017; Galan et al., 2018), no post hoc bioinformatic procedures can identify false negatives. Two primary strategies have been adopted to maximize the taxonomic coverage of primer sets used in metabarcoding studies. One strategy consists in designing “universal” primer sets that target DNA of all taxa in the clade of interest (e.g., Leray et al., 2013; Clarke, Soubrier, Weyrich, & Cooper, 2014; Rennstam Rubbmark, Sint, Horngacher, & Traugott, 2018). However, in silico or in vivo (sensu Alberdi et al., 2018; i.e., based on environmental samples) tests demonstrated that many “universal” primer sets were far from having perfect taxonomic coverage (e.g., Deagle, Jarman, Coissac, Pompanon, & Taberlet, 2014; Alberdi et al., 2018; but see Elbrecht & Leese, 2017a). The other strategy consists in using a cocktail of primer sets targeting either the same locus (e.g., Gibson et al., 2014; Corse et al., 2017) or distinct loci (e.g., Kaunisto, Roslin, Sääksjärvi, & Vesterinen, 2017; Olmos‐Pérez, Roura, Pierce, Boyer, & González, 2017; Devloo‐Delva et al., 2018).

Previously, we developed a benchtop‐to‐desktop workflow to analyze the diet of an invertivorous fish. We evaluated the taxonomic coverage of two primer sets targeting the 5′ end of the barcode region of the cytochrome *c* oxidase subunit I gene (COI) in silico, in vitro (using tissue‐derived invertebrate DNA), and in vivo (using fish feces; Corse et al., 2017). Here, we introduce a new primer set designed to reduce false negatives. We combined it with the previous two primer sets using a “one‐locus‐several‐primer‐sets” (OLSP) strategy to assay environmental samples. We first assessed the taxonomic coverage of the three primer sets with in silico analyses of current available barcodes. We then conducted an in vivo evaluation of the taxonomic and haplotypic diversity coverage and complementarity of the three primer sets on environmental DNAs (eDNA) obtained from a variety of materials as follows: feces from invertivores living in brackish water, freshwater, and terrestrial habitats, and spider webs. Finally, we brought new insights into the diet of the brackish water fish *Pomatoschistus microps* (Krøyer, 1838) and of the African freshwater fish *Epiplatys infrafasciatus* (Günther, 1866), and we assessed the biodiversity of invertebrates trapped in spider webs from the Amazon rainforest.

## 2 MATERIAL AND METHODS

### 2.1 Primer design

In the workflow we developed previously (see Corse et al., 2017), we used two primers sets (MFZR and ZFZR; for details, see Table 1) for the detection of invertebrate diversity in fish fecal samples. We aimed here to improve our workflow by developing a new COI primer set that covers an additional diversity of prey species and haplotypes. In this perspective, we manually designed a new forward primer (LepLCO) and two degenerate reverse primers (McoiR1 and McoiR2; for details, see Supporting Information Appendix S1). These primers produced ~150 bp amplicons in the 5′ end of the COI barcode region, that largely overlap with the sequences amplified by the primers sets MFZR and ZFZR. Additionally, we evaluated the reverse primer MLepF1‐rev (Brandon‐Mong et al., 2015) when used with LepLCO (see Table 1 for details).

**Table 1:** Primers and primer sets evaluated in silico, in vitro*,* and in vivo in this study

Primer set	Primer name	Forward (F)/Reverse (R)	Sequence (5′−3′)	Reference	Evaluation of primer sets:		
					**In silico**	**In vitro**	**In vivo**
MFZR	Uni‐Minibar‐F1	F	TCCACTAATCACAARGATATTGGTAC	Meusnier et al. (2008)	Yes	Yes	Yes
	ZBJ‐ArtR2c	R	WACTAATCAATTWCCAAATCCTCC	Zeale, Butlin, Barker, Lees, and Jones (2011)			
ZFZR	ZBJ‐ArtF1c	F	AGATATTGGAACWTTATATTTTATTTTTGG	Zeale et al. (2011)	Yes	Yes	Yes
	ZBJ‐ArtR2c	R	WACTAATCAATTWCCAAATCCTCC	Zeale et al. (2011)			
LFCR	LepLCO	F	RKTCAACMAATCATAAAGATATTGG	This study	Yes	Yes	Yes
	McoiR2	R	CCBCCRATTAWAATKGGTATHAC	This study			
LepLCO/McoiR1	LepLCO	F	RKTCAACMAATCATAAAGATATTGG	This study	Yes	Yes	No
	McoiR1	R	AATCCBCCRATTAWAATKGGTAT	This study			
LepLCO/MLepF1‐rev	LepLCO	F	RKTCAACMAATCATAAAGATATTGG	This study	Yes	Yes	No
	MLepF1‐rev	R	CGTGGAAAWGCTATATCWGGTG	Brandon‐Mong et al. (2015)			
fwhF1/fwhR1	fwhF1	F	YTCHACWAAYCAYAARGAYATYGG	Vamos et al. (2017)	Yes	No	No
	fwhR1	R	ARTCARTTWCCRAAHCCHCC	Vamos et al. (2017)			
MG‐LCO1490‐MiSeq/MG‐univR‐MiSeq	MG‐LCO1490‐MiSeq	F	ATTCHACDAAYCAYAARGAYATYGG	Galan et al. (2018)	Yes	No	No
	MG‐univR‐MiSeq	R	ACTATAAARAARATYATDAYRAADGCRTG	Galan et al. (2018)			

### 2.2 In silico evaluation of primers

The taxonomic coverage of seven primers sets (of which three include newly designed primers; see Table 1) was estimated for 36 taxa (see Supporting Information Table S1) according to the approach implemented in primerminer‐0.11 (Elbrecht & Leese, 2017b). Briefly, for each taxonomic group, all COI sequences were downloaded from the NCBI nt database using COi, CO1, COXi, COX1 as keywords as well as all COI sequences from the BOLD database (www.boldsystems.org; Ratnasingham & Hebert, 2007) in February 2017. Sequences were clustered with VSEARCH v2.9.0 (Rognes, Flouri, Nichols, Quince, & Mahé, 2016) implemented in primerminer using a 3% dissimilarity threshold to avoid redundancy, and then, the majority consensus sequences of the clusters were aligned. Only sequences that completely covered the primer annealing site were considered. The number of consensus sequences varied among taxa from 1 to 2075 (median 32; considered taxa listed in Supporting Information Table S1). primerminer then provided a penalty score: We used the default value (i.e., 120) to determine whether the consensus sequences should be successfully amplified (score < 120) or not (score > 120) by the primers.

### 2.3 In vitro selection of primers

The primers sets LepLCO/McoiR1, LepLCO/McoiR2 (LFCR), and LepLCO/MLepF1‐rev were assayed using the DNA from 16 distinct specimens of four invertebrate species (further details in Supporting Information Appendix S1). We selected the primer set that unambiguously amplified all 16 samples: LFCR.

### 2.4 In vivo evaluation of primers

The taxonomic coverage of MFZR, ZFZR, and LFCR was empirically evaluated through metabarcoding of 107 eDNA samples (Table 2). DNA was extracted from samples following Corse et al. (2017), and the safety measures to prevent cross contamination are described by Monti et al. (2015). Our analysis also included extraction, aerosol, PCR, and tag negative controls (respectively, *Text*, *Tpai*, *T_PCR_*, and *Ttag* in Corse et al., 2017) and two different mock community samples (*Tpos1* and *Tpos2*) as positive controls (Table 3). Samples and controls were amplified by PCR in triplicate using tagged primer sets MFZR, ZFZR, and LFCR, resulting in a total of nine separate PCRs per sample/control. The tags used were 11–13 nucleotide long sequences differentiated by at least three different nucleotides (for details see: Corse et al., 2017). These tags were added on the 5′ end of the primers (12 and 8 distinct tags were used to label forward and reverse primers, respectively). Amplicons were then processed and sequenced on an Illumina MiSeq v3 platform with the paired‐end 250‐nucleotide technology. HTS data were then filtered using the variant‐centered (clustering‐free) approach detailed in Corse et al. (2017) (see Supporting Information Figure S1), which minimizes the amount of errors and false positives/negatives. Briefly, reads were merged and assigned to samples based on forward and reverse tag combinations. After trimming off tags and primers, identical reads were pooled into variants (dereplication). Based on positive and negative controls, read counts for each variant in each sample were used to define thresholds for a series of filtering steps to eliminate low‐frequency noise (LFN; sensu De Barba et al., 2014; Supporting Information Figure S1). LFN filtering steps were optimized to keep all variants within mock samples (*Tpos1* and *Tpos2*) and to eliminate unexpected variants, that is, any variants in negative controls, variants in mock samples that are not part of the mock community, variants in eDNA samples that were unexpected given the source habitat, for example, DNA of freshwater organisms in a brackish water sample (see below). Throughout the filtering procedure, the reproducibility of variants was ensured by (a) eliminating PCR replicates that had a high Renkonen distance to other replicates within the same sample and (b) by retaining only variants that were present in at least two different PCR replicates of the same sample (for a similar approach, see Alberdi et al., 2018; Galan et al., 2018). Variants from different primer sets that were identical in their overlapping regions (~130 bp) were combined into contigs. The taxonomic assignment of each variant/contig was conducted (a) automatically using the lowest taxonomic group approach (Corse et al., 2017) and (b) manually using BOLD systems. When assignment levels were insufficient or when the two approaches conflicted, a third assignment method was conducted by building phylogenetic trees and/or considering biogeographic information (Corse et al., 2017). The combined use of these three assignment approaches led to a final taxonomic assignment for each variant/contig. After an initial round of filtering and taxonomic assignment, the variants that were unexpected given their source habitat were identified. Based on the frequency of these unexpected variants, a second round of filtering was run using adjusted LFN thresholds that maximize the elimination of unexpected variants (Supporting Information Figure S1). Finally, to standardize the evaluation of coverage and complementarity between primer sets, all validated variants/contigs were clustered into molecular operational taxonomic units (MOTUs) based on a 3% divergence threshold using complete‐linkage clustering.

**Table 2:** Environmental samples analyzed in this study

Predator	Number of samples	Environmental sample	Region, habitat	Country	Locality	Coordinates	Sampling date
*Zingel asper *(fish)	46	Feces	Palearctic, freshwater	France	Durance River	N 44°18′54″, E 5°55′31″	October‐2014
*Pomatoschistus microps *(fish)	15	Feces	Palearctic, brakish water	France	Vaccarès Lagoon	N 43°33′14″, E 4°30′21″	May‐2012
*Pomatoschistus microps *(fish)	14	Feces	Palearctic, brakish water	France	Prévost Lagoon	N 43°31′32″, E 3°54′46″	May‐2012
Unknow bat species	1	Feces	Palearctic, terrestrial	France	Rancogne	N 45°41′48″, E 0°24′12″	August‐2015
*Myotis nattereri* (bat)	2	Feces	Palearctic, terrestrial	France	Vilhonneur	N 45°40′50″, E 0°25′11″	June‐2015
*Pipistrellus pipistrellus *(bat)	1	Feces	Palearctic, terrestrial	France	Vilhonneur	N 45°40′50″, E 0°25′11″	July‐2015
*Eptesicus serotinus* (bat)	1	Feces	Palearctic, terrestrial	France	Vilhonneur	N 45°40′50″, E 0°25′11″	July‐2015
*Miniopterus schreibersii* (bat)	4	Feces	Palearctic, terrestrial	France	Rancogne	N 45°41′48″, E 0°24′12″	August‐2015
*Barbastella barbastellus *(bat)	1	Feces	Palearctic, terrestrial	France	Rancogne	N 45°41′48″, E 0°24′12″	August‐2015
*Rhinolophus euryale* (bat)	1	Feces	Palearctic, terrestrial	France	Rancogne	N 45°41′48″, E 0°24′12″	August‐2015
*Rhinolophus ferrumequinum* (bat)	1	Feces	Palearctic, terrestrial	France	Saint‐Bonnet‐sur‐Gironde	N 45°21′16″, W 0°39′34″	December‐2015
*Myotis emarginatus* (bat)	1	Feces	Palearctic, terrestrial	France	Saint‐Bonnet‐sur‐Gironde	N 45°21′16″, W 0°39′34″	December‐2015
*Epiplatys infrafasciatus *(fish)	6	Feces	Equatorial, freshwater	Cameroon	Lokoundje River	N 3°4′41″, E 10°24′02″	May‐2012
Unknown spider species	3	Spider web	Equatorial, terrestrial	French Guiana	Monkey Mountain, SW Kourou	N 5°4′25″, W 52°42′4″	July‐2016
Araneomorphae (spider)	5	Spider web	Equatorial, terrestrial	French Guiana	Monkey Mountain, SW Kourou	N 5°4′25″, W 52°42′4″	July‐2016
Pholcidae (spider)	2	Spider web	Equatorial, terrestrial	French Guiana	Monkey Mountain, SW Kourou	N 5°4′25″, W 52°42′4″	July‐2016
*Micrathena schreibersi* (spider)	3	Spider web	Equatorial, terrestrial	French Guiana	Monkey Mountain, SW Kourou	N 5°4′25″, W 52°42′4″	July‐2016

**Table 3:** Community composition of mock samples used as positive controls (*Tpos1* and *Tpos2*)

Positive controls		Species	DNA concentration (ng µl^−1^)	Taxonomic group	Corresponding variant/contig
Tpos1	Tpos2	*Ephemerella ignita*	0.2	Ephemeroptera	contig_0019
Tpos1	Tpos2	*Hydropsyche modesta*	0.2	Trichoptera	contig_0054
Tpos1		*Oligoneuriella rhenana*	0.2	Ephemeroptera	contig_0077
Tpos1		*Eisenia andrei*	0.2	Oligochaeta	contig_0417
Tpos1	Tpos2	*Chironomus riparius*	0.2	Diptera	LFCR_006421
Tpos1		*Dinocras cephalotes*	0.2	Plecoptera	LFCR_009263
Tpos1		*Phoxinus cf. phoxinus*	0.2	Cypriniformes	MFZR_010307
	Tpos2	*Hydropsyche instabilis*	0.2	Trichoptera	contig_0027
	Tpos2	*Gammarus pulex*	0.2	Crustacea	contig_0038
	Tpos2	*Planorbarius corneus*	0.2	Gastropoda	contig_0053
	Tpos2	*Velia saulii*	0.2	Heteroptera	contig_0055
Tpos1	Tpos2	*Zingel asper*	0.8	Perciformes	LFCR_005960

Note

Mock samples were based on pooled DNAs, which were extracted from individual invertebrate specimens.

Since all predators in this study were mainly invertivores, we assumed that most macroinvertebrates (and vertebrates) constituted relevant prey and referred to them as “Macrometazoans.” Hence, items that most likely result from passive ingestion or secondary predation such as microinvertebrates (e.g., Amoebozoa, Acari, Tardigrada, Rotifera), diatoms, algae, and plants, as well as potential parasites (e.g., Acanthocephala, Nematoda), were excluded from the analyses (for a similar approach, see Hardy et al., 2017).

To evaluate the coverage and complementarity of the three primer sets, we looked at their performance in detecting different prey groups from the 107 eDNA samples at various taxonomic levels as follows: Phylum, Class, Order, Family, MOTU, and variant. The coverage of each primer set was estimated through the coverage ratio *Bc* (Ficetola et al., 2010). Here, *Bc* corresponds to the ratio between the number of taxa detected in samples by a given primer set and the total number of taxa detected by all three primer sets. The complementarity (*Com*) of the primer sets was assessed by dividing the number of prey items detected by one primer set only by the total number of detected taxa. In addition, we measured samples’ pairwise differences in diet composition between primer sets (*Wsd *for within‐sample dissimilarity) with pairwise the Bray–Curtis index (Bray & Curtis, 1957). Finally, pairwise Bray–Curtis dissimilarities between samples were also calculated (*Bsd* for between‐sample dissimilarities).

### 2.5 Diet analyses

Diet analyses were performed using the Minimal Number of Individuals (MNI; White, 1953) matrix of prey items. The MNI is a semiquantitative statistic that corresponds to the number of distinct variants and/or contigs validated in each sample (see Corse et al., 2017). We further assessed the taxonomic resolution of our diet metabarcoding dataset as a function of predator type, habitat, and geographic location by calculating the mean identification resolution index (*IR*; see Zarzoso‐Lacoste et al., 2016) calculated as detailed in Corse et al. (2017): A score was attributed for each variant/contig based on the taxonomic level of its final taxonomic assignation (i.e., Species = 6, Genus = 5, Family = 4, Order = 3; Class = 2, Phylum = 1, Kingdom or NA = 0), and then, a mean score among the variants was calculated for each sample.

## 3 RESULTS

### 3.1 In silico evaluation of primers

**Figure 1:**
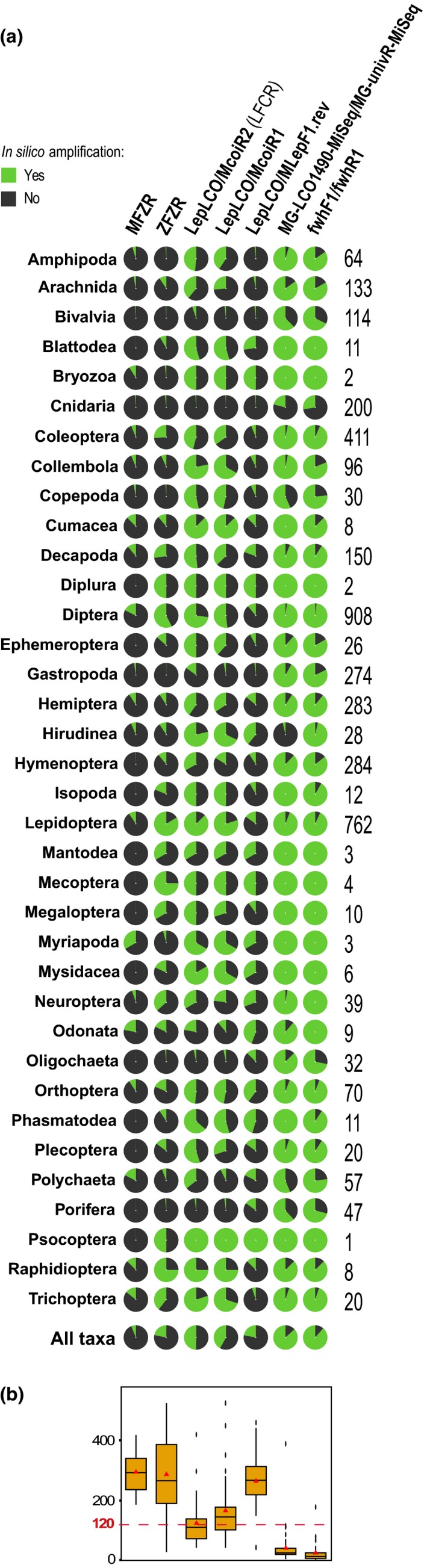
In silico evaluation of primer set performance using primerminer. (a) Primer set performance for each taxon in pie charts (green = success; black = failure). On the right, the median number of sequences per taxon used for in silico evaluation of primer sets. (b) Distribution of the median primerminer penalty scores for each primer pair. Mean values are indicated by a triangle within boxplots

The in silico analysis revealed that theoretical amplification success was generally quite low and varied strongly across primer sets and target taxa (Figure 1). The average in silico amplification success for MFZR, ZFZR, and LFCR was 8%, 34%, and 53%, respectively, and their median primerminer penalty scores were 292,8, 372,8, and 124,7 (Supporting Information Tables S1 and S3). Hence, LFCR is expected to perform better than ZFZR and MFZR in terms of taxonomic coverage.

### 3.2 Metabarcoding data

The raw dataset was gathered from 13 distinct MiSeq runs. After preprocessing, the dataset consisted of 15.1 millions (M) of reads that correspond to the three PCR replicates of 107 eDNA samples, 39 negative controls, and 26 mock community samples. Filtering thresholds were determined by run, based on variant occurrence and frequencies (filtering parameters including LFN thresholds are reported in Supporting Information Table S4) and then applied separately for each run. After filtering, 195 variants were validated for MFZR, 237 for ZFZR, and 186 for LFCR. These corresponded to 0.4% of the variants initially identified as COI, but 73% of the reads identified as COI (11.0 M validated reads). After combining the MFZR, ZFZR, and LFCR variants, 168 contigs and 212 variants were obtained. At the end of the filtering process, all the initial eDNA samples contained by at least one variant or contig. Only one negative control (TnegPai1_DNA11; see Supporting Information Table S5) was not eliminated: Two variants assigned to a marine species (*Anapagurus hyndmanni*; contig_0365 and contig_0745; Supporting Information Table S5) were validated, though not found in any other environmental samples. All seven variants expected in mock samples *Tpos1* and *Tpos2* were retrieved. In most of the mock samples, however, one or two extra variants were also validated (contig_0238, contig_0124, MFZR_000591; Supporting Information Table S5). As these variants were absent from all other samples/controls, we suggest these arose from organisms ingested by or attached to one of the invertebrate individuals used to build the mock samples. By recovering all of the taxa of the mock communities (*Tpos1* and *Tpos2*) in all the different runs, we assumed that we minimized random fluctuations, making our samples comparable between runs.

**Figure 2:**
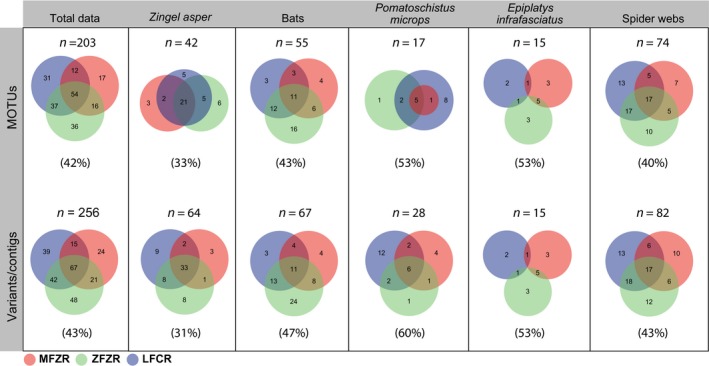
Macrometazoan MOTUs and variants obtained from environmental samples using each primer set. The mean biodiversity complementarity (*Com*) is in brackets

A total of 256 distinct Macrometazoan variants/contigs were obtained from eDNA samples (178 were detected by ZFZR, 127 by MFZR, and 163 by LFCR) corresponding to 203 Macrometazoan MOTUs after clustering (143 MOTUs were detected by ZFZR, 99 by MFZR, and 134 by LFCR; Figure 2).

### 3.3 In vivo evaluation of the coverage and complementarity of primer sets

**Figure 3:**
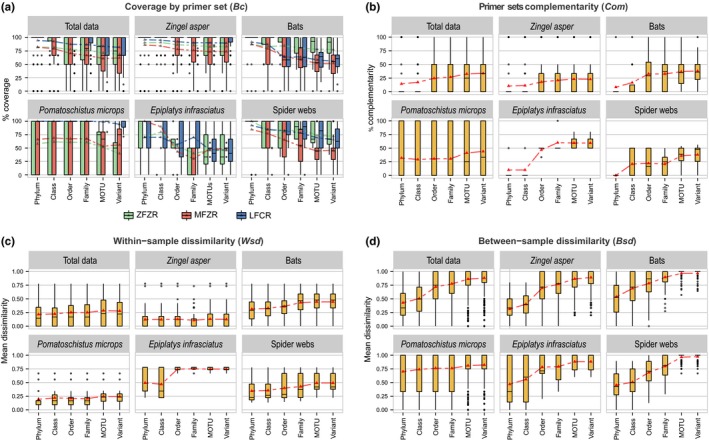
Coverage complementarity and dissimilarity of primer sets. (a) Coverage (*Bc*) of primer sets, (b) complementarity of primer sets (*Com*), (c) within‐sample dissimilarity (*Wsd*), and (d) dissimilarity between environmental samples (*Bsd*). Mean values are indicated by a triangle within boxplots. Dissimilarities are Bray–Curtis dissimilarities calculated from Minimal Number of Individuals (MNIs). Only Macrometazoans are considered here

Across the whole dataset, the coverage (*Bc*) differed between primer sets (Kruskal–Wallis test; *Χ* ^2^ = 77.23, *df* = 2, *p* < 10^−15^) with LFCR performing 1.1 and 1.2 times better than ZFZR and MFZR, respectively. Mean *Bc* of primer sets decreased at finer taxonomic level: *Bc_ZFZR_* ranged from 71% to 81%, *Bc_MFZR_* from 61% to 81%, and *Bc_LFCR_* from 81% to 93% (Figure 3a). However, for all three primer sets, coverage varied sharply across the different predator categories. For example, ZFZR displayed the highest *Bc* for bat samples (82% on average) while it displayed the lowest *Bc* for *P. microps* samples (55% on average). LFCR exhibited the highest *Bc* in *P. microps* samples (98% on average). For the three primer sets, the *Bc* at variant level was similar to that obtained at the MOTU level. However, we observed that primer set success could vary between variants within MOTUs. Distinct variants within the same MOTU were differentially detected by primer sets for 47% of the 36 Macrometazoan MOTUs for which more than one variant was detected. For instance, in *P. microps* samples, five different sequences formed the MOTU cluster 119 (Mysidae) of which one variant (contig_0001) was amplified by all primer sets, one by MFZR and ZFZR primer sets only (contig_0468), and three by MFZR only (MFZR_000169, MFZR_004854, MFZR_010569; Supporting Information Table S5; Appendix 2).

The complementarity (*Com*) of primer sets depended both on the taxonomic level and on the predator type (Figure 3b). The mean *Com* index differed significantly between the taxonomic levels (Kruskal–Wallis test; *Χ* ^2^ = 88.97, *df* = 5, *p* < 10^−15^) and steadily increased from phylum (mean *Com* = 14.8%) to variant (mean *Com* = 33.4%; Figure 3b). Although the increase of complementarity from phylum to variant is general, its order of magnitude differed sharply across predators (e.g., *Zingel asper* vs. *E. infrafasciatus*; Figure 3b). Furthermore, even at the MOTU and variant levels, the mean *Com* differed significantly across predator types (Kruskal–Wallis test; *Χ* ^2^ = 26.15, *df* = 4, *p* = 0.00003 for MOTUs; *Χ* ^2^ = 25.02, *df* = 4, *p* = 0.00005 for variants).

Similarly to the coverage and to the complementarity, the within‐sample dissimilarity (*Wsd*) depended on the predator (Kruskal–Wallis test; *Χ* ^2^ = 205.11, *df* = 4, *p* < 10^−15^) with the lowest mean values observed for *Z. asper* (*Wsd* = 0.12) and *P. microps* (*Wsd* = 0.22) and the highest ones observed for *E. infrafasciatus* (*Wsd* = 0.65; Figure 3c). Furthermore, for all predators, within‐sample dissimilarity tended to increase with greater taxonomic resolution of prey, especially for spider webs, *E. infrafasciatus,* and bat samples. Moreover, *Wsd* values were very close to (or even exceeded in the case of *E. infrafasciatus* samples) the values of pairwise dissimilarity indexes between samples (Figure 3d).

### 3.4 Taxonomic identification and resolution

**Figure 4:**
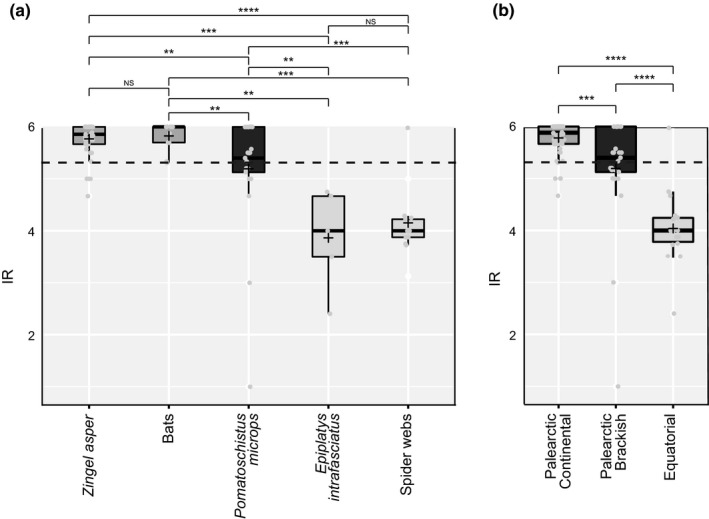
Identification resolution (*IR*) index of environmental samples. (a) For each predator type, and (b) by habitat/geographic location. Mean values are indicated by “+”. Significance levels of pairwise Kruskal tests are indicated on top: n.s.: nonsignificant; *p < 0.05; **p < 0.01; ***p < 0.001; ****p < 0.0001. Only Macrometazoans are considered here

The calculation of *IR* allowed for a standardized comparison between samples concerning their taxonomic resolution. Across all environmental samples, the mean *IR* for Macrometazoans was 5.33 (±0.91). *IR* significantly differed between predator types, habitats, and geographic locations (Figure 4). The mean *IR* values were close to the maximal value for bats and *Z. asper* (*IR* = 6), which corresponded to an average taxonomic assignment of variants to species level. The mean *IR* was only slightly lower for *P. microps* samples, while the taxonomic resolution of Macrometazoans in the two types of equatorial samples (*E. infrafasciatus* feces and spider webs) were close to family level (mean *IR* = 4.05 ± 0.69).

### 3.5 Diet results

**Figure 5:**
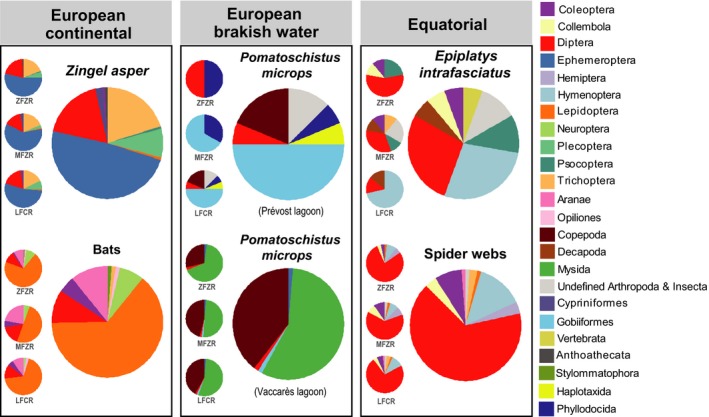
Macrometazoan taxa detected in fecal and spider web samples. The proportions of prey items are based on the cumulative Minimal Number of Individuals (MNIs). Proportions for each primer set are presented to the left of the main pie chart. Only Order‐level taxonomic assignments are presented

The Macrometazoans detected in the 107 environmental samples covered a wide taxonomic array of invertebrates and included some vertebrate prey as well (Figure 5). The proportion of cumulative MNI for non‐Macrometazoans represented <20% of the total dataset and varied from ~10% (*P. microps*) to ~40% (*E. infrafasciatus*) of the total (Appendix 1).

**Figure 6:**
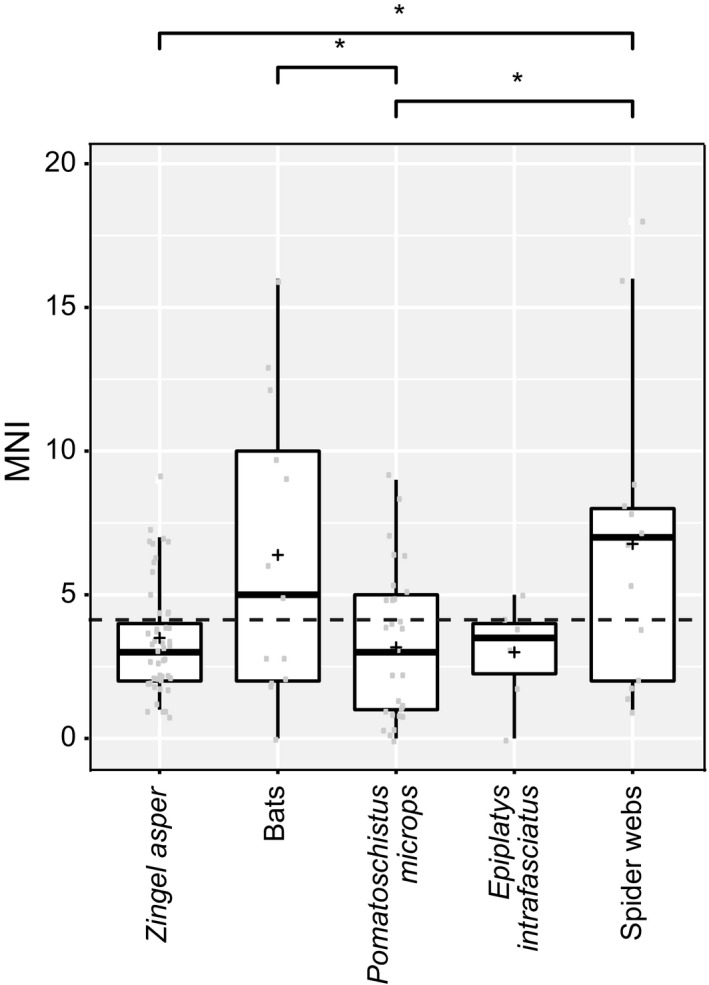
Minimal Number of Individuals (MNI) detected in fecal and spider web samples. Significance levels of pairwise Kruskal tests on top (*p < 0.05). Only Macrometazoans are considered here

Predator DNA was not detected in any of the 46 *Z. asper *fecal samples. The mean MNI per sample was 3.50 ± 1.97 (Figure 6). Macrometazoan prey of *Z. asper* was aquatic invertebrates such as Ephemeropera (8 MOTUs), Trichoptera (6 MOTUs), Diptera (13 MOTUs), and Gammaridae (4 MOTUs; Supporting Information Table S5). We also detected DNA from benthic fish species (*Barbus barbus* and *Barbatula *sp.) and allochthonous prey (undetermined Nymphalidae). More than one variant was detected for ~30% of Macrometazoan MOTUs. The MOTU assigned to *Baetis fuscatus* was the most diverse (six distinct variants) and was also the most frequently detected prey (37% of the total MNI).

Bat DNA was detected in 5 of 13 bat fecal samples. The mean MNI per sample was 6.38 ± 5.09 (Figure 6). Prey DNAs were mainly assigned to flying insects as follows: Lepidoptera (29 MOTUs), Diptera (8 MOTUs) and Neuroptera (3 MOTUs). We also detected ground‐dwelling invertebrates such as Coleoptera (4 MOTUs) and the white‐lipped snail (*Cepea hortensis*). Lepidoptera were the primary prey while Diptera, Araneae, and Coleoptera constituted secondary prey. This pattern is similar to the prey composition estimated by Galan et al. (2018) for a similar set of bat species.

Predator DNA was detected in all 29 *P. microps *fecal samples. The mean MNI per sample was 3.17 ± 2.59 (Figure 6). Our data revealed sharp differences among sampling locations in *P. microps* diet. The *P. microps* from Prévost Lagoon were mainly piscivorous, predating their syntopic sister species *Pomatoschistus minutus* (detected in 40% of fecal samples), whereas the *P. microps* from Vaccarès Lagoon ingested mainly Mysidae (5 MOTUs) and Copepoda (3 MOTUs). Only one fish variant (assigned to *Pomatoschistus *sp.; one occurrence in fecal sample P16) was detected in Vaccarès.

Predator DNA was detected in all six *E. infrafasciatus *fecal samples. The mean MNI per sample was 3.00 ± 1.78 (Figure 6). Macrometazoan DNA detected in *E. infrafasciatus* fecal samples was a mix of aquatic organisms such as crustacean (Atyidae) and terrestrial invertebrates such as ants (Formicidae) and springtails (Collembola), with the ratio of potential allochthonous prey being ~60%. Interestingly, a variant assigned to ray‐finned fishes (Actinopterygii) was detected in one fecal sample.

No DNA of spiders was detected from any spider web samples. The mean MNI per web sample was 6.76 ± 5.34 (Figure 6). Macrometazoans detected in spider webs were mostly flying insects with a high diversity detected for Diptera (52 MOTUs, including 24 Cecidomyiidae MOTUs) and Hymenoptera (9 MOTUs). We also detected variants assigned to Coleoptera, Trichoptera, and Collembola.

## 4 DISCUSSION

### 4.1 In vivo tests are essential to determine primer sets

The discrepancies in coverage of primer sets that we observed among in silico, in vitro, or in vivo analyses illustrates the difficulties in predicting the performance of primer sets in complex and degraded eDNA mixtures. In metabarcoding studies, in silico evaluation of PCR performance is often used as a key step for designing and/or selecting primers (e.g., Ficetola et al., 2010; Clarke et al., 2014; Kartzinel & Pringle, 2015; Elbrecht & Leese, 2017a; Elbrecht & Leese, 2017b). However, the actual amplification success of these primers on DNA mixtures or on complex environmental samples often differs substantially from in silico predictions (Alberdi et al., 2018; Corse et al., 2017; Morales & Holben, 2009). Such differences may be explained by two main causes. First, the database used for in silico analyses was built from publicly available sequences, which is likely to miss a lot of the species present in the environmental samples being analyzed. Second, in silico analyses are highly sensitive to the algorithms used for estimating primer performance and overlook other important factors influencing PCR success such as PCR inhibitors in varying amounts and competition among DNA strains for primers, often due to a difference in affinity. Our results and those from Alberdi et al. (2018) emphasize that although in silico and in vitro analyses are important in narrowing down the optimal primer sets (e.g., Elbrecht & Leese, 2017a; Piñol, Senar, & Symondson, 2019), in vivo tests are essential and the ultimate step for selecting primers that maximize biodiversity coverage and minimize PCR biases.

### 4.2 The use of multiple primers is key to finely describe species diversity

Our three primer sets were shown to perform equally well in detecting higher taxonomic ranks (phyla and classes). On the contrary, there were significant differences in coverage at finer taxonomic levels (e.g., more than 40% of the MOTUs were detected by one primer set only). Interestingly, complementarity slightly increased at the taxonomic level “variant” revealing that even variants belonging to the same MOTUs were not equally detected by all three primer sets. Consequently, the more desirable is a fine taxonomic resolution (MOTUs or variants), the more important is the use of multiple primer sets for revealing greater diversity and decrease false negatives. Furthermore, some primer sets will be more suitable than other depending on the predator. For example, ZFZR has the best coverage for Lepidoptera (*Bc* = 87%; Appendix 2). When sequencing the diet of bats that eat in majority Lepidoptera (Figure 5), this primer set will be better as it detects more MOTUs than the other primer sets (Figure 2). LFCR, on the other hand, has the best taxonomic coverage for Crustacea (*Bc* = 86%; Appendix 2) and, consequently, covered 94% of MOTUs and 79% of variants detected in *P. microps* feces. Overall, our results highlight the need to use multiple primers sets when one wants to tackle finely prey diversity and especially when the prey communities can be highly variable. It should be noted that some authors proposed to use one single highly degenerated primer set as an alternative (Elbrecht & Leese, 2017a, Piñol et al., 2019).

### 4.3 The “one‐locus‐several‐primer‐sets” strategy

The COI gene is a locus of choice when studying invertebrate diversity and is largely used for this. However, the high variability that makes the COI a powerful marker consequently makes truly universal primer sets hard to design. Yet, several recent attempts, that involved highly degenerated primers, appeared very promising (Elbrecht & Leese, 2017a; Vamos et al., 2017; Galan et al., 2018; see also Figure 1), and comparative in vivo tests (sensu Alberdi et al., 2018) would be desirable to confirm the broad applicability of these highly degenerated primers for exhaustively recovering the biodiversity in environmental samples.

Alternatively, we followed an OLSP strategy (used in Corse et al., 2017; but see also Gibson et al., 2014) targeting the COI gene using three distinct primer sets (MFZR, ZFZR and LFCR). Initially developed for studying the diet of *Z. asper*, we applied this strategy to other types of invertivores to fully evaluate the applicability of our approach and found that each primer set detected at least some biodiversity that was hidden from the other primer sets (Figure 2). In some cases, we observed critical differences among primer sets: The estimates of the ingested prey communities were sharply different depending on the primer set considered for *E. infrafasciatus* or for *P. microps* from Prévost Lagoon (Figure 5). Consequently, whether using one primer set or another is expected to considerably influence the qualitative interpretation of the trophic behavior of these two species. In fact, the differences in prey composition (*Wsd*) for a same sample obtained with different primers were very close or higher than that observed between samples (*Bsd*), the former representing technical noise, and the later representing a biological signal (Figure 3c,d), hence confirming the conclusions by Alberdi et al. (2018). The use of several primer sets targeting the same taxa should compensate the high between‐primer set variance in biodiversity detection and expectedly yield a more comprehensive picture of the prey diversity. In addition to maximizing coverage, the OLSP approach produces fully comparable homologous sequences that will facilitate the evaluation of biodiversity at variant level. In our case, all three primer sets target the same locus (the 5′ end of the COI gene) and reads were merged into contigs when overlapping sequences (~130 bp) were identical such that the three different datasets could be either compared together or merged into a single dataset.

The use of amplicon sequence variants instead of MOTUs in metabarcoding analyses was recently suggested to improve and refine measurements of biodiversity (Callahan, McMurdie, & Holmes, 2017; Wares & Pappalardo, 2016). In fact, variant information was used to quantify intraspecific genetic diversity (Elbrecht, Vamos, Steinke, & Leese, 2018; Pedro et al., 2017; Sigsgaard et al., 2016) and was shown to approximate taxa abundance reliably for diet analyses (Corse et al., 2017). However, only robust and reliable datasets free from possible false positives and artifacts can allow such use of variant level information. Consequently, use of variant information in quantitative or semiquantitative ways will require reliable and robust bioinformatics filtering procedures of HTS data (see Corse et al., 2017).

### 4.4 Public databases and the taxonomic assignment of prey

Both the confidence and the resolution of taxonomic assignment procedures are highly dependent on the completeness of reference sequence databases (e.g., Gibson et al., 2014; Porter et al., 2014; Devloo‐Delva et al., 2018). In this study, we illustrated that the accuracy of taxonomic assignment of prey depended on the environment of the predators (Figure 4b). Palearctic samples (feces from *Z. asper*, *P. microps* and bats) displayed a high taxonomic resolution of prey with most variants assigned to species. On the contrary, samples from the tropics (feces of *E. infrafasciatus* from Africa and spider webs from South America) displayed a much lower taxonomic resolution generally corresponding to family level (Figure 4b). In megadiverse environments and regions, such as equatorial environments, our ability to identify sequences precisely (e.g., at species level) is indeed strongly restricted by relatively incomplete public databases (e.g., Cowart et al., 2015; Beng et al., 2016; Lopes et al., 2017; Stat et al., 2017). Our results therefore illustrate the role of completeness of public databases and the importance in investing into species inventories to better understand food web and species interactions.

### 4.5 Insights into the diet of *Pomatoschistus microps* and *Epiplatys infrafasciatus*

To our knowledge, our OLSP approach using a variant‐based filtering procedure is the first to reveal the trophic ecology of *P. microps* and *E. infrafasciatus *with metabarcoding data.

*Pomatoschistus* species are valuable models for studying adaptation in coastal and estuarine environments (e.g., Pampoulie, Chauvelon, Rosecchi, Bouchereau, & Crivelli, 2001; Larmuseau, Vancampenhout, Raeymaekers, Houdt, & Volckaert, 2010) and historical processes of colonization in the Mediterranean (e.g., Tougard, Folly, & Berrebi, 2014). The trophic interaction of *P. microps* with other sympatric gobies, such as the sand goby *P. minutus*, is an important topic of investigation (e.g., Salgado, Cabral, & Costa, 2004; Leitão et al., 2006). Before our study, the knowledge on the diet of *P. microps* was studied using gut‐content analysis (e.g., Magnhagen & Wiederholm, 1982). The preys we detected were congruent to those previous observations (i.e., mostly zooplankton and benthic organisms) and included Copepoda, Mysidae, Chironomidae, and Annelida (Figure 5; Supporting Information Table S5). In addition, we detected other *Pomatoschistus* species, which would confirm interspecies predation as already reported in the *Pomatoschistus* genus (Hamerlynck & Cattrijsse, 1994). Interestingly, important differences were observed between the two sampled localities: *P. microps* from Vaccarès Lagoon fed mostly on Mysidae whereas *P. microps* from Prévost fed mostly on *P. minutus*. The heterogeneous level of interspecific predation may be explained by the scarcity of other *Pomatoschistus* species in Vaccarès Lagoon (Pampoulie et al., 2001).

*Epiplatys* species inhabit lentic rivers, usually near the surface under the leaves of plants (e.g., Romand & Morgalet, 1983). Diet of *Epiplatys* are mainly composed of aquatic insects (Guma'a, 1982; Ndome & Victor, 2002) although terrestrial organisms such as Formicidae may also represent important preys (Romand & Morgalet, 1983). This was the case here, with ~60% of *Epiplatys* prey being composed of terrestrial organisms such as Formicidae, Collembola, and Psocoptera and only 15% of preys being composed of unambiguously aquatic organisms (Figure 5; Supporting Information Table S5). Collembola may constitute allochthonous prey fallen from riparian vegetation or, given their small size (2–3 mm), secondary prey for *E. infrafasciatus* (i.e., ingested by their primary prey such as Formicidae). These results are consistent with *E. infrafasciatus* top‐mouth position that favors capture of prey at the water surface, notably allochthonous ones. We also detected ~25% of algae and fungi in *E. infrafasciatus* feces, suggesting that this species could also forage aufwuchs communities as reported for *E. senegalensis* (Ndome & Victor, 2002). Although only based on six fecal samples, our results suggest that *E. infrafasciatus* exhibits quite versatile foraging behaviors, exploiting both pelagic and benthic habitats and feeding on both allochthonous and aquatic prey.

### 4.6 The capture of biodiversity by spider webs

Spider webs represent a potential noninvasive source of DNA for conservation, ecology, and management studies (Blake, McKeown, Bushell, & Shaw, 2016; Xu, Yen, Bowman, & Turner, 2015). They are able to trap a part of the local arthropod biodiversity present in natural (even pristine), agricultural, or urban habitats and may serve as “biodiversity capsules” (sensu Boyer, Cruickshank, & Wratten, 2015), especially for flying insects. To date, DNA extracted from spider webs enabled the detection of both the predator and its prey using diagnostic PCR under controlled conditions (Blake et al., 2016; Xu et al., 2015). We here present the first attempt to analyze the biodiversity captured by natural spider webs using metabarcoding. Among our environmental samples, spider webs from the equatorial forest of French Guiana displayed the highest biodiversity by far displaying the highest mean MNI (Figure 6) and the highest MOTU diversity (Supporting Information Tables S6). The main taxa detected were flying insects, especially Diptera (52 MOTUs) and Hymenoptera (9 MOTUs), but also some Coleoptera, Trichoptera, Lepidoptera, and Hemiptera. However, contrary to previous studies using diagnostic PCR (Blake et al., 2016; Xu et al., 2015), we were unable to detect the DNA of the spiders that spun the webs even though our primer sets were able to detect a non‐negligible part of Arachnida in bat fecal samples (Figure 5). Regardless, we illustrate that spider webs do act as natural traps of biodiversity, and we confirmed that they are a promising tool for invertebrate diversity assessments when combined with the power of HTS (as suggested by Xu et al., 2015).

## 5 CONCLUSIONS

We demonstrated the lack of predictability of both the coverage and complementarity of individual primer sets. Although in silico and in vitro (i.e., based on DNA extracted from isolated organisms) evaluations are useful to narrow down a subset of primer sets for metabarcoding studies (e.g., Elbrecht & Leese, 2017a; Piñol et al., 2019), in vivo evaluation of primers based on a subset of environmental samples is critically informative for minimizing false negatives before conducting larger scale analyses. Furthermore, we formalized the “one‐locus‐several‐primers” (OLSP) strategy that directly addresses primer set coverage biases to minimize false negatives. This strategy produces largely overlapping and comparable sequences, which cannot be achieved when targeting different loci, and facilitates the use of genetic diversity information contained within metabarcoding datasets. However, we emphasize the importance of stringent variant‐based filtering procedures to validate HTS data (Callahan et al., 2016; Corse et al., 2017) before genetic information of metabarcoding datasets can be used to estimate (semi‐)quantitative diversity indices. Our workflow, combining the OLSP strategy and stringent variant‐based filtering, can be easily adapted and extended to other loci and other applications for studying biodiversity through metabarcoding.

## CONFLICT OF INTEREST

None declared.

## AUTHOR CONTRIBUTIONS

E.C., V.D., C.T., and C.C.Y.X. conceived and designed the study. E.C., V.D., G.A., F.D.M.M., C.F.B.B., J.F.A., R.C., L.Z., D.D., and C.T. collected the samples. G.A. identified invertebrates morphologically. E.C., V.D., and E.M. developed the method. E.C. and C.T. performed the experiments. E.C., E.M., and V.D. analyzed the data. E.C., V.D., and E.M. wrote the paper. All authors drafted the paper.

## Data Availability

Supplementary data deposited in Dryad (https://doi.org/10.5061/dryad.2ck7120) include the unfiltered HTS data from the MiSeq runs used in this paper, as well as the sequence alignment generated with primerminer for in silico evaluation of metabarcoding primer sets.

## APPENDIX 1 Proportion of Macrometazoan and non‐Macrometazoan variants in fecal and spider web samples

The proportion of prey items are based on the cumulative Minimal Number of Individuals (MNIs).
