# Supplementary figures and images for: One‐locus‐several‐primers: A strategy to improve the taxonomic and haplotypic coverage in diet metabarcoding studies
Source: Ecol Evol. 2019 Mar 18;9(8):4603–20. doi: 10.1002/ece3.5063 (PMC6476781; doi:10.1002/ece3.5063)

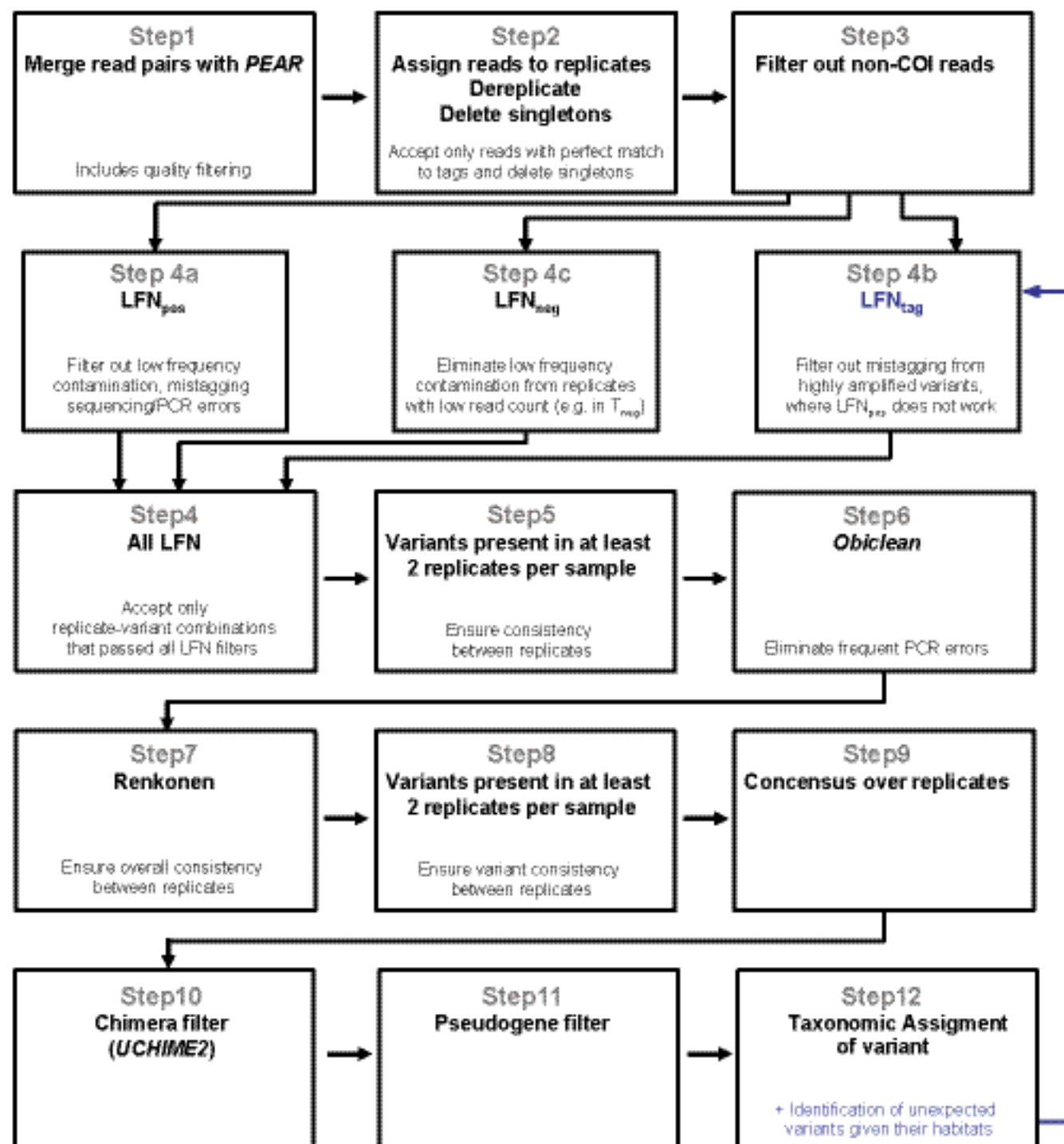

Figure S1: High-throughput sequencing filtering pipeline. For further details, see Corse et al. 2017.

Supplement: Supplementary file 1 [file ECE3-9-4603-s001.pdf]
