# Supplementary material for: One‐locus‐several‐primers: A strategy to improve the taxonomic and haplotypic coverage in diet metabarcoding studies
Source: Ecol Evol. 2019 Mar 18;9(8):4603–20. doi: 10.1002/ece3.5063 (PMC6476781; doi:10.1002/ece3.5063)
Supplement: Supplementary file 3 [file ECE3-9-4603-s003.docx]

**Supplementary File 1. Primer design and *in vitro* selection**

The workflow we initially developed (published in Corse et al. 2017) was based on two primer sets (MFZR and ZFZR; for details, see: Table 1 and Corse et al., 2017), which target the same minibarcode located in the 5’ end of the COI gene. Some previous *in vitro* tests showed that PCR amplification was poor or failed for some specimens of *Chironomius riparius* (Diptera), *Dinocras cephalotes* (Plecoptera), *Gammarus pulex* (Amphipoda) and *Ecdyonurus venosus* (Ephmeroptera). However, PCR amplification and DNA sequencing were successful for all specimens when using a cocktail of primers (CK4; Corse et al., 2017), which amplifies the entire COI barcode and includes the forward primers LepF1 (Hebert et al., 2004) and LCO1490 (Folmer et al., 1994) forward primers. Thus, we created a new forward primer LepLCO (5’-RKTCAACMAATCATAAAGATATTGG-3’) from the consensus of primers LepF1 and LCO1490. For the reverse primer, COI sequences of the four species above were aligned, and two degenerate primers were manually designed based on the sequence variability of the four taxa. These reverse primers produced ~150 bp amplicons when used with LepLCO: McoiR1 (5’-AATCCBCCRATTAWAATKGGTAT-3’), McoiR2 (5’- CCBCCRATTAWAATKGGTATHAC-3’). Additionally, we evaluated the reverse primer MLepF1-rev (Brandon-Mong et al., 2015) when used with LepLCO (Table 1).

To ensure that the primers sets LepLCO/McoiR1, LepLCO/McoiR2, and LepLCO/MLepF1-rev amplified the target species (i.e. *C. riparius*, *D. cephalotes*, *E. venosus*, and *G. pulex*), they were assayed using the DNA of four specimens of each species (for a total of 16 samples) following the PCR conditions described in Corse et al. (2017) for the CK4 primer set. We selected the primer pair that unambiguously amplified all 16 samples: LepLCO/McoiR2 (i.e. LFCR).
